# Influence of bodily resonances on emotional prosody perception

**DOI:** 10.3389/fpsyg.2022.1061930

**Published:** 2022-12-08

**Authors:** Garance Selosse, Didier Grandjean, Leonardo Ceravolo

**Affiliations:** ^1^Neuroscience of Emotion and Affective Dynamics Lab, Department of Psychology, University of Geneva, Geneva, Switzerland; ^2^Swiss Center for Affective Sciences, University of Geneva, Geneva, Switzerland

**Keywords:** emotional prosody, perception, embodiment, bodily resonances, interoception

## Abstract

**Introduction:**

Emotional prosody is defined as suprasegmental and segmental changes in the human voice and related acoustic parameters that can inform the listener about the emotional state of the speaker. While the processing of emotional prosody is well represented in the literature, the mechanism of embodied cognition in emotional voice perception is very little studied. This study aimed to investigate the influence of induced bodily vibrations—through a vibrator placed close to the vocal cords—in the perception of emotional vocalizations. The main hypothesis was that induced body vibrations would constitute a potential interoceptive feedback that can influence the auditory perception of emotions. It was also expected that these effects would be greater for stimuli that are more ambiguous.

**Methods:**

Participants were presented with emotional vocalizations expressing joy or anger which varied from low-intensity vocalizations, considered as ambiguous, to high-intensity ones, considered as non-ambiguous. Vibrations were induced simultaneously in half of the trials and expressed joy or anger congruently with the voice stimuli. Participants had to evaluate each voice stimulus using four visual analog scales (joy, anger, and surprise, sadness as control scales).

**Results:**

A significant effect of the vibrations was observed on the three behavioral indexes—discrimination, confusion and accuracy—with vibrations confusing rather than facilitating vocal emotion processing.

**Conclusion:**

Over all, this study brings new light on a poorly documented topic, namely the potential use of vocal cords vibrations as an interoceptive feedback allowing humans to modulate voice production and perception during social interactions.

## Introduction

Oral communication is critical for a social animal such as the human being, both through semantic meaning of words and non-verbal parameters. Voice ‘prosody’ refers to changes in these acoustic parameters that complement the information or even add new information given by language (Scherer and Oshinsky, 1977; Risberg and Lubker, 1978; Laukka et al., 2005; Grandjean et al., 2006), and represents the so-called melody of the voice, which is related to pitch temporal variations, with other information such as spectral fluctuations related to timbre. Acoustic parameters (e.g., energy) are mainly shaped by the mechanisms of vocalization production, including mainly breathing, phonation, and articulation, which are themselves modulated by emotion (Banse and Scherer, 1996). Therefore, emotional prosody conveys information about the affective state of the speaker, and has a key role in the regulation of social interactions (Sauter and Eimer, 2010; Pell and Kotz, 2011). Scherer’s adaptation of Brunswik’s lens model (Brunswik, 1956; Scherer, 2003) is an illustration of the processes behind the expression and the perception of emotional prosody from a speaker to a listener. Indeed, the listener perceives the modified parameters in the voice of the speaker and can make subjective attribution of his or her emotional state, often influenced by contextual information (Grandjean et al., 2006). However, emotional prosody perception relies upon several mechanisms. Indeed, hearing emotional vocalizations activates processes of embodied simulation that allow us to understand more accurately the emotion of the speaker (Hawk et al., 2012). Simulation involves the integration of motor, sensory and affective representations of the speaker’s emotional state. These processes of embodied simulation are more globally part of the concept of embodied cognition.

According to the theories of embodied cognition, mental representations are constructed through the interaction of motor, sensory and affective systems (Niedenthal, 2007). Indeed, the body seems to intrinsically constrain and modulate our cognitive processes (Foglia and Wilson, 2013). As a matter of fact, gesturing grounds people’s mental representations in action (Beilock and Goldin-Meadow, 2010) and in a similar way, access to autobiographical memories is improved in a body position that is congruent between encoding and retrieval (Dijkstra et al., 2007). In the light of evolutionary processes, understanding other individuals and their intentions is crucial in social interactions (Stevens and Fiske, 1995; Kaschak and Maner, 2009). In a study with a monkey, Rizzolatti and Craighero (2004) observed neurons in the ventral premotor cortex that were found to be activated for actions performed by the monkey, but also when observing the performance of that action by another individual. This system of so-called “mirror neurons” is a mechanism that is presumed to be involved in the understanding of the actions of others, and therefore in a more global way in the processes of social organization. However, the presence of this category of neurons in humans is still debated (Turella et al., 2009), even if empirical findings compatible with its existence were reported (Mukamel et al., 2010).

There are several mechanisms involved in the perception of emotions. In the visual modality, three distinct mechanisms have been observed. The first one is the visual analysis of facial parameters and is dominant when decoding prototypical facial expressions. The second one is the conceptual analysis of the emotion taking into account available knowledge about the emitter and contextual information such as the social situation (Niedenthal, 2008). The last one—and the main topic of the present study—is the embodied simulation, defined as the process by which a facial expression triggers “a simulation of a state in the motor, somatosensory, affective, and reward systems that represents the meaning of the expression to the perceiver” (Niedenthal et al., 2010; p. 418). Several authors showed that the perception of body states in others produces a body mimicry in the observer (Dimberg, 1982; Maxwell et al., 1985; Neumann and Strack, 2000; Barsalou et al., 2003; Korb et al., 2010; Moody and McIntosh, 2011). Recently, many studies support the hypothesis that facial mimicry reflects the sensorimotor simulation of an observed emotion rather than a simple muscular reproduction of an observed facial expression (Hess and Fischer, 2014; Borgomaneri et al., 2020). According to the Simulation of Smiles Model (SIMS model; Niedenthal et al., 2010), the embodied simulation intervenes mainly when facial expressions are not prototypical and visual analysis of facial parameters is no longer sufficient, but also when the individual is particularly interested in understanding the individual facing him or her. However, this model addresses processes related to facial recognition of emotion, i.e., the visual modality, while our study focuses on the auditory modality. We have nevertheless considered this model for our hypotheses because we believe that some of these processes are related to emotional processing in a cross-modal way. Indeed, it has been shown that emotion processing is a complex mechanism with multimodal sensory integration (Campanella and Belin, 2007; Collignon et al., 2008). Moreover, multimodal integration has also been investigated in the field of embodied cognition. Studies showed that individuals use their body and their senses as different sources of information and that they integrate them to communicate more effectively (Price and Jewitt, 2013; Mills et al., 2022). However, no study has investigated the integration of bodily resonances in the perception of emotional prosody in a multimodal context.

The present study focuses on the process of cognition embodied in the auditory perception of emotions. Several studies have revealed that passive listening of voice signals triggered activations in motor brain regions similar to those solicited during production (Fadiga et al., 2002; Watkins et al., 2003; Wilson et al., 2004). Another study showed a facilitation of language comprehension when the primary motor areas controlling the lips or tongue were transcranially stimulated for sounds produced by either muscle, respectively (D'Ausilio et al., 2009). However, research in this area remains scarce and more work is needed to understand more deeply the different kinds of mechanisms that can be involved in embodied emotional prosody perception both at the behavioral and brain level.

In the present study, we investigated the potential role of bodily resonances as a type of interoceptive feedback during the perception of vocal emotions. During voice production, vibrations originating in the vocal cords are emitted. They propagate through the skin and organic tissues of the speaker (Švec et al., 2005; Munger and Thomson, 2008). The frequency of these bodily resonances corresponds mainly to the fundamental frequency of the tone produced (Sundberg, 1992). These bodily resonances were measured by accelerometers (Nolan et al., 2009), namely sensors that measure the mechanical vibrations of solid bodies or by laser Doppler vibrometers (Kitamura and Ohtani, 2015). These measures can be only quantified at the surface of the skin, and are virtually insensitive to sound (Švec et al., 2005), which makes them particularly useful for studying bodily resonances. Numerous studies have explored the locations of these vibrations, and it seems that regions like the nasal bone, the zygomatics, the temples, above the upper lip and in the upper neck are the most relevant ones to consider (Sundberg, 1992; Munger and Thomson, 2008; Nolan et al., 2009). In the domain of singing, singers speak of ‘chest register’ and ‘head register’ to distinguish the pitches of voices (Sundberg, 1977, 1992). The chest register is characterized by more energy in low-frequency, whose resonances are perceived more in the chest while the head register corresponds more to higher frequencies, whose resonances are perceived more strongly in the skull. The sensation of bodily resonances would represent a stable and dynamic feedback for the speaker of his or her own voice (Sundberg, 1992), since they are not disturbed by the usual acoustic characteristics of the environment. Anatomically, the receptors for perceiving movements and thus body vibrations are mainly the different kinds of mechanoreceptors (Caldwell et al., 1996). For example, it is likely that the sensation of certain bodily resonances by Pacini’s corpuscles can serve as a useful non-auditory signal for the voluntary control of phonation with respect to low frequencies (Gauffin and Sundberg, 1974; Sundberg, 1983, 1992). Lylykangas et al. (2009) have highlighted the possibility of successfully using vibrotactile feedback as relevant information for speed-regulation messages. In the same way, Tuuri et al. (2010) compared audio and tactile feedback coming from the same voice stimuli as speed-regulation messages and they observed that the information was transmitted similarly across modalities.

To our knowledge, no study has investigated the role of the bodily vibrations in the auditory perception of emotions, which is the purpose of the present study. The main hypothesis is that the vibrations of the vocal cords, necessary for the production of vocalized sounds, would constitute an interoceptive feedback that can influence the auditory perception of emotions in an embodied perspective of perception. As the SIMS model specifies that the effect of embodied simulation is observed during the perception of non-prototypical emotional stimuli, our study uses ambiguous emotional voice stimuli. We focus on the modulation of auditory perception, with emotional voice stimuli presented at different intensities (three distinct sound pressure levels), the lowest one being considered as less obvious or more ambiguous and therefore more difficult to perceive. In our study, a vibration was induced only in half of the trials and it was always congruent—same speaker, vocal production, and emotion—with the emotional vocalization presented auditorily. Three behavioral indices were computed. The discrimination index indicates the extent to which the emotion expressed is distinguished from others by the participant. The confusion index measures the extent to which the participant perceives emotions other than the one expressed. Finally, accuracy is a dichotomous measure that indicates whether the emotion recognized as the dominant one by the participant is the one expressed. The task was designed to test two main hypotheses. First, it was hypothesized that vibrations would have an impact on judgments of emotional vocalizations. More specifically, we predicted that, when vibrations were induced, (i) discrimination would be higher, (ii) confusion would be lower, and (iii) accuracy would be higher compared to the condition without vibration. Second, these effects were assumed to be greater for the less intense stimuli, i.e., low intensity emotional vocalizations, than for medium or high intensity emotional vocalizations. Indeed, less intense stimuli would convey unclear information and should therefore be the modalities in which information is sought elsewhere, according to the SIMS model, for instance *via* embodied simulation that is here characterized by the induced vibrations.

## Materials and methods

### Participants

Twenty-one healthy volunteers were recruited amongst psychology students from the University of Geneva (twenty females, one male; *M_Age_* = 24.27; *SD_Age_* = 5.62). All participants were at least 18 years old, reported normal hearing and no neurologic or psychiatric history. Participants gave informed and written consent for their participation in the experiment. The study was approved by the local ethics committee in accordance with ethical and data security guidelines of the University of Geneva and conducted according to the declaration of Helsinki.

### Materials

Participants were presented with emotional voice stimuli through headphones (HD25, Sennheiser, DE) with Matlab (The Mathworks Inc., Natick, MA, United States), using the Psychophysics Toolbox extensions (Brainard, 1997; Pelli, 1997; Kleiner et al., 2007). The stimuli were presented while a fixation cross was displayed on the screen. Voice stimuli were pseudo-sentences consisting of alternating vowels and consonants in order to avoid the bias of semantic knowledge of words from the French language. The two phrases recorded from actors were “nekal ibam soud molen!” and “koun se mina lod belam?.” We used 32 different stimuli from 16 actors, 16 expressing joy, and 16 expressing anger. One stimulus expressing joy was excluded from the analyses, due to a significantly higher confusion as compared to the other stimuli (Supplementary Figure 1). Overall, the emotions expressed were well recognized: 92.39% of anger voice stimuli were categorized as anger while 73.49% of joy voice stimuli were categorized as joy (Supplementary Table 1). Voice stimuli were played at three intensities: low, medium, or high, which varied in amplitude (Supplementary Figure 2) and then loudness but not on other acoustic parameters. Anger and joy voice stimuli showed no difference in amplitude and loudness, but they varied in pitch, roughness, and spectral parameters (Supplementary Table 2). Intensity, emotion and actor were randomly distributed across trials. Voice and vibration stimuli come from the validated Geneva Multimodal Expression Portrayals (GEMEP) database (Bänziger et al., 2012).

**Figure 1:**
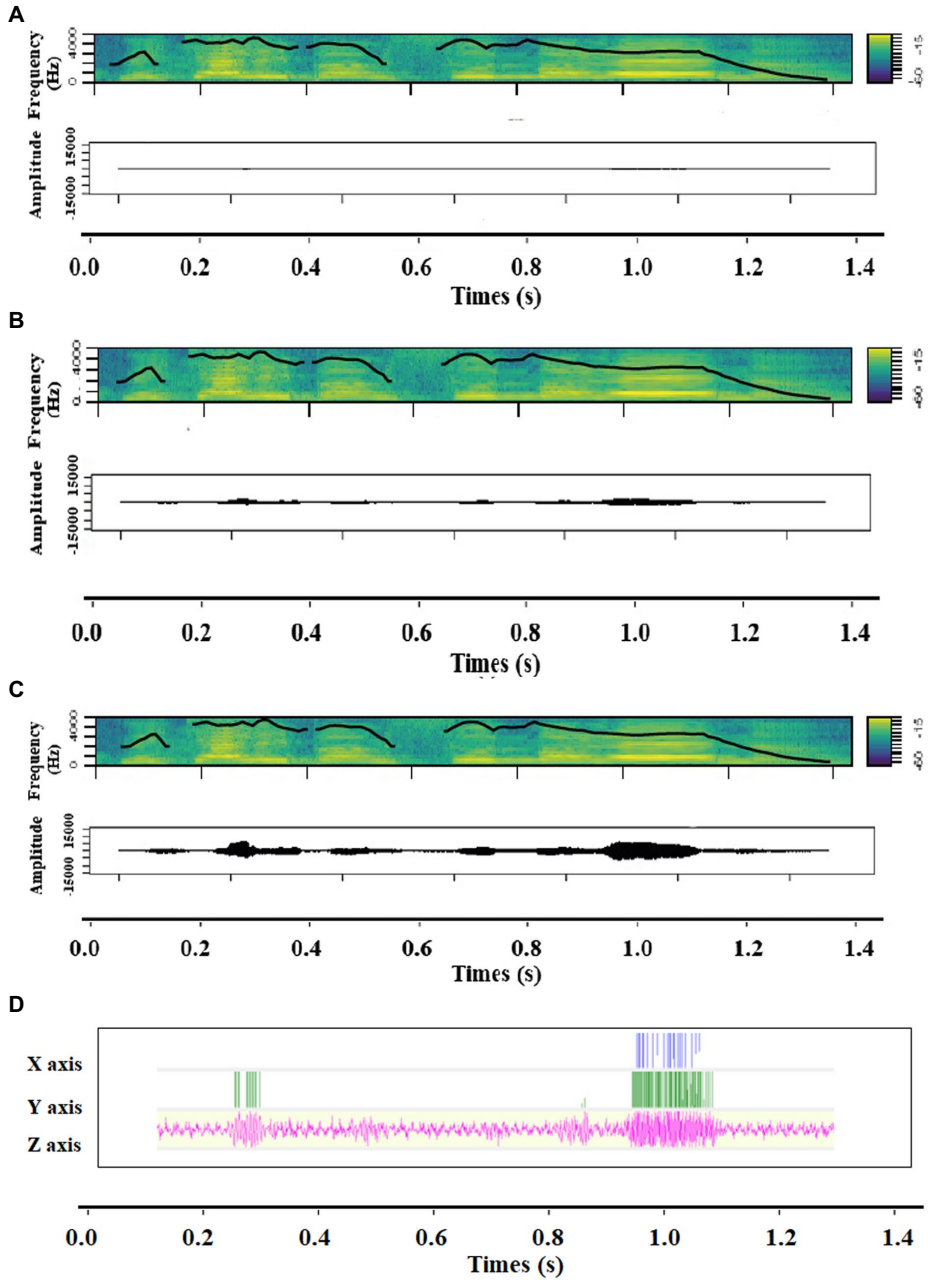
Spectrograms including contour of fundamental frequency and Amplitude time series of **(A)** Low intensity, **(B)** Medium intensity and **(C)** High intensity, as well as **(D)** the accelerometer signal of the vibration of a sound expressing anger.

**Figure 2:**
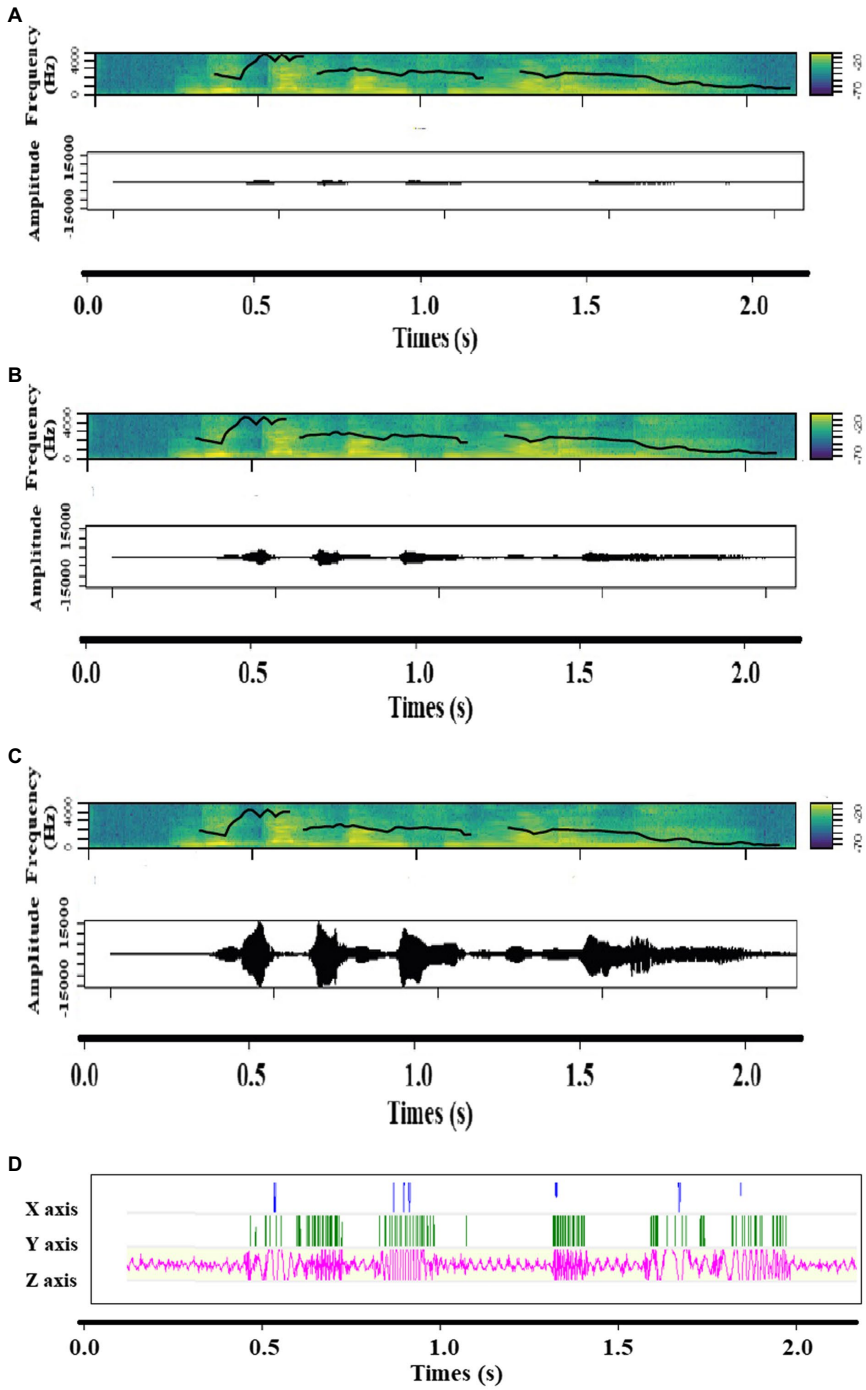
Spectrograms including contour of fundamental frequency and Amplitude time series of **(A)** Low intensity, **(B)** Medium intensity and **(C)** High intensity, as well as **(D)** the accelerometer signal of the vibration of a sound expressing joy.

**Figure 3:**
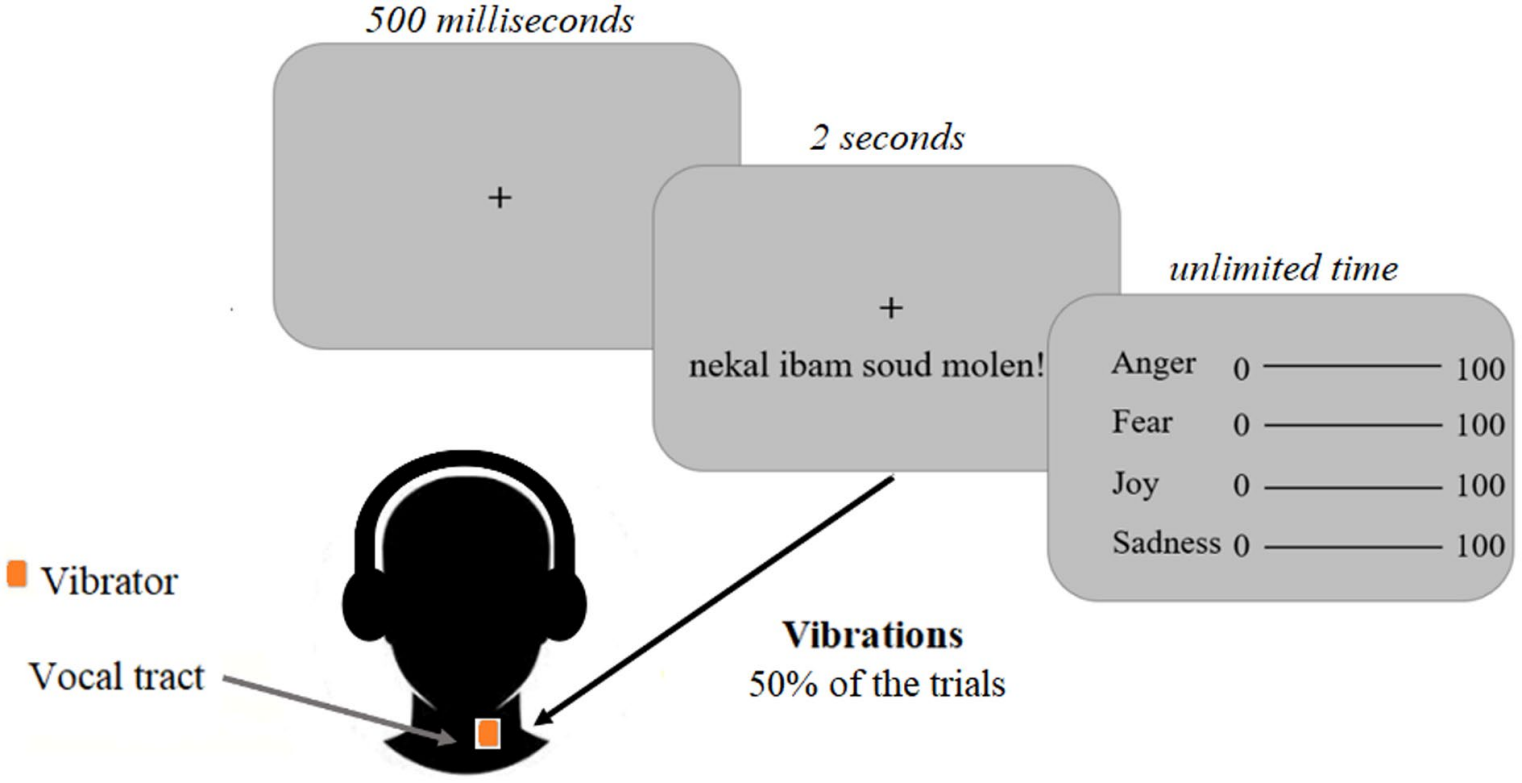
Experimental design of Study 1. Participants were instructed to focus on a black central fixation cross displayed on a grey screen for five hundred milliseconds. Emotional voice stimuli were presented through headphones for 2 s. Vibrations were induced at the same time in half of the trials and matched the voice stimuli. The vibrator was located close to the vocal cords. After the voice stimuli, visual analogue scales were displayed indicating that the participants had to evaluate the emotion expressed in the voice stimuli, without time constraints.

Bodily resonances were induced in the form of vibrations expressing either joy or anger in half of the trials while no vibrations were induced in the remaining half of the trials as a control measure. Two conditions of bodily resonances were therefore implemented (absent, present). In each trial with vibration, voice stimuli and vibration were congruent—the input stimulus was the same—except for the fact that voice stimuli varied in intensity (Low, Medium, High, Figures 1, 2) while the vibrations’ intensity stayed constant (High-intensity condition). Bodily resonances were created by transmitting emotional voice stimuli to a device that mechanically converted these sound waves into vibrations. The acoustic parameters of these vibrations were those of the emotional voice stimuli used, and therefore varied depending on the emotion expressed. Speaker identity was identical for each combination of voice and vibration. No difference in amplitude and loudness were found significant between anger and joy vibrations, but they varied in pitch, roughness, and spectral parameters (Supplementary Table 2). The vibrator’s (BC-10, Ortofon, DK) dimensions are 13.5*29.5*18.0 millimeters, it weighs 16.5 grams, has a sensitivity of 118 decibels, a total harmonic distortion of 1.5%, an impedance of 15 ohm and sensibility ranging from 100 to 1,000 Hz with a sampling rate of 1,000 Hz. It was positioned close to the vocal cords (Figure 3), taped on the left side to the laryngeal prominence, also known as Adam’s apple, with kinesiology tape. To create the vibration, the vibrator transforms the waveform of an input sound into mechanical energy, here a vibratory signal.

### Task procedure

The experiment comprised four blocks each including 48 randomly presented stimuli. The complete duration of the experiment including the installation of the vibrator equipment was approximately 50 mins per participant. A trial consisted of the display of a centered fixation cross screen for five hundred milliseconds, followed by the presentation of a voice stimulus for 2 s while the same centered fixation cross screen was displayed (Figure 3). This voice stimulus was accompanied or not by a vibration from the vibrator (50% of the trials, counterbalanced). Then, a second screen allowed the participant to evaluate the voice stimulus using four visual analog scales (joy, anger, and surprise, sadness as control scales; Figure 3). For each emotion, the person had to move a cursor using the mouse and each scale ranged from 0 to 100 (0 for an emotion that was not expressed and 100 for an emotion that was strongly expressed) to indicate the degree to which this emotion was represented according to him/her. This step was not time-limited. The total value of the four cursors was not supposed to be 100, each measure was independent from the others and participants were informed there were no good or bad responses.

### Statistical analysis

The results consisted of four measures of emotion for each trial, each between 0 and 100. The target emotion was joy for half the trials, and anger for the other half. Two indices from the four cursors were computed to run the analyses. First, the discrimination index (DI) is an indication of how well the target emotion is recognized compared to the three other possibilities of the visual analogue scales. It was calculated as follows:

***DI = target emotion – sum of the three irrelevant emotions.***

For each trial, the sum of the three irrelevant emotions was subtracted from the value given to the emotion expressed (joy or anger). It therefore potentially ranges from -300 to 100, a higher score meaning a better recognition of the target emotion.

Another index was computed to only take into account the impact of the three emotions that were not expressed in the voice stimulus: the confusion index (CI). It was calculated as follows:

***CI = sum of the three irrelevant emotions.***

For each trial, the sum of the three irrelevant emotions is calculated. This index potentially ranges from 0 to 300, a higher score meaning a greater score for one of many irrelevant emotions and therefore a higher confusion. Four other emotion-specific confusion indices were calculated to assess the degree of confusion of each stimulus for each possible emotion present in the visual analogue scales (Supplementary Table 3).

Moreover, the accuracy was also taken into account in the analyses. It was calculated as follows:

***Accuracy = target emotion/ (sum of the four emotions).***

The accuracy ranges from 0 to 1, a higher score meaning a greater accuracy in the recognition of the target emotion.

Linear Mixed Models (LMM) with a 2*3*2 design were performed in R (R Core Team, version 1.4.1103, 2020) to analyze these three indices in response to “Emotion” (anger, joy), “Intensity” (low, medium and high) and “Vibration” (absent, present), respectively. “Gender of the actor” and “Block” were also included as factors in the models as control measures. This statistical model allowed the integration of random effects (Venables and Dichmont, 2004; Bolker et al., 2009), namely the ‘identifier of the subjects’ in this study. The effect sizes of each model were computed and labelled according to the thresholds defined by Cohen (1988; *r* effects: small ≥ .10, medium ≥ .30, large ≥ .50). Two different effect sizes, marginal and conditional R^2^, were presented as recommended and defined by Nakagawa and Schielzeth (2013): “*Marginal R^2^ is concerned with variance explained by fixed factors, and conditional R^2^ is concerned with variance explained*.”

## Results

Two Linear Mixed Models and a Generalized Linear Mixed Model with a 2*3*2 design were performed to, respectively, analyze each measure (discrimination/confusion measure) and the accuracy in response to the following factors: Emotion (joy, anger), Intensity (low, medium, high) and Vibration (present, absent).

### Discrimination index

**Figure 4:**
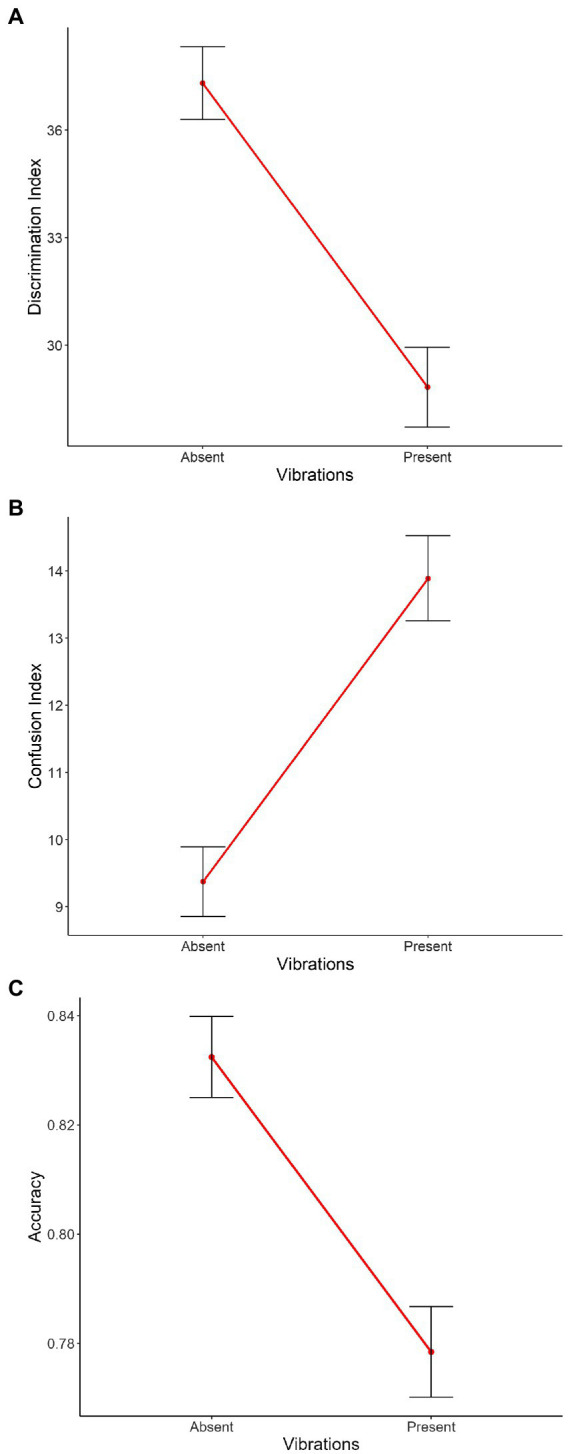
Main effect of Vibration on the mean of **(A)** the discrimination index, **(B)** the confusion index, and **(C)** the accuracy. On the X axis, each point represents Vibration (i.e., absent, present). Error bars indicate the standard error of the mean.

**Figure 5:**
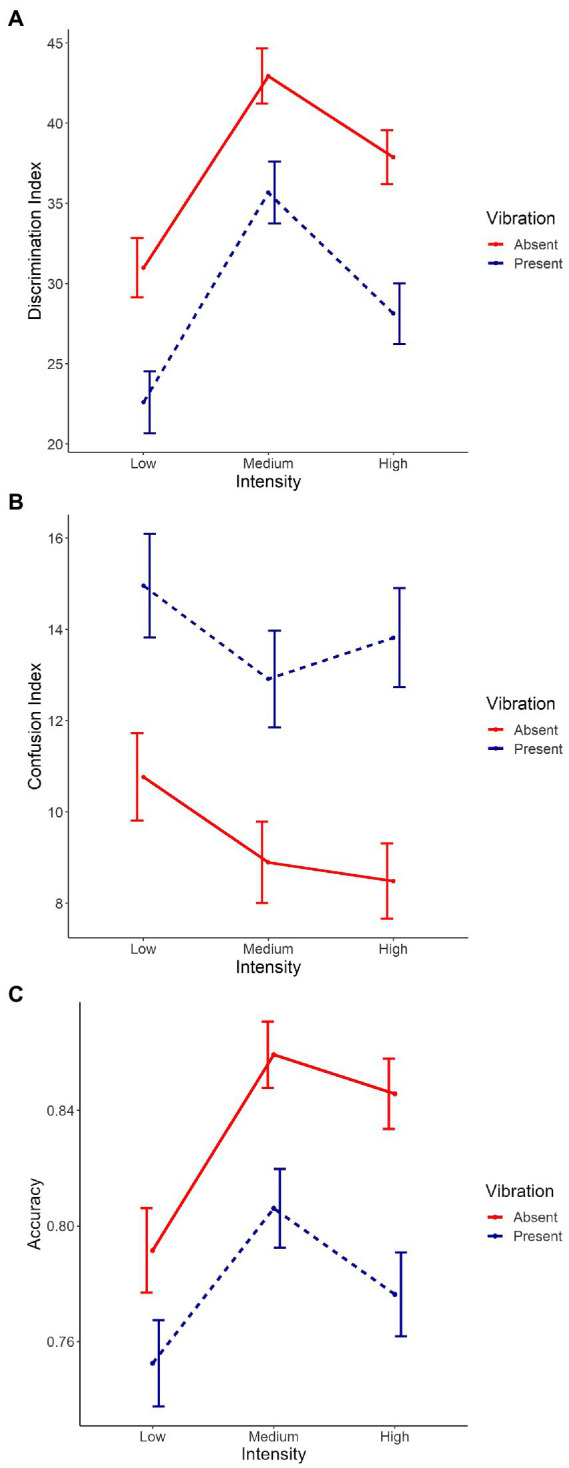
Interaction between Vibration and Intensity on the mean of **(A)** the discrimination index, **(B)** the confusion index, and **(C)** the accuracy. Intensity conditions are represented on the X axis (i.e., low, medium and high). Each line represents Vibration (i.e., absent, present). Error bars indicate the standard error of the mean.

The main effects of Vibration [*χ^2^*(1) = 28.04, *p* < 0.001, Figure 4A], Intensity [*χ^2^*(2) = 60.54, *p* < 0.001, Supplementary Figure 3A] and Emotion [*χ^2^*(1) = 421.20, *p* < 0.001, Supplementary Figure 4A] were significant, with absent vibrations associated with higher discrimination compared to present vibrations [Absent: *m* = 37.3, *sd* = 42.6; Present: *m* = 28.8, *sd* = 48.2; *χ^2^*(1) = 7.12, *p* < 0.001], and joy associated with lower discrimination compared to anger [Joy: *m* = 18.3, *sd* = 51.5; Anger: *m* = 46.8, *sd* = 18.3; *χ^2^*(1) = 27.65, *p* < 0.001]. Planned contrasts were performed to investigate the effect of Intensity and the three conditions were significantly different from each other with discrimination scores from highest to lowest: medium, high, and low intensities [Low: *m* = 26.7, *sd* = 46.5; Medium: *m* = 39.2, *sd* = 45.4; High: *m* = 32.8, *sd* = 44.6; low vs. medium: *χ^2^*(1) = −12.89, *p* < 0.001, low vs. high: *χ^2^*(1) = −6.53, *p* < 0.001, medium vs. high: *χ^2^*(1) = 6.36, *p* < 0.001]. The interaction between Emotion and Intensity was significant as well [*χ^2^*(2) = 7.46, *p* < 0.05, Supplementary Figure 6A]. The other double interactions [Emotion*Vibration: *χ^2^*(1) = 0.31, *p* = 0.58, Supplementary Figure 5A; Intensity*Vibration: *χ^2^*(2) = 0.49, *p* = 0.78, Figure 5A] and the triple interaction [*χ^2^*(2) = 1.17, *p* = 0.56] were not significant. The effect size of this model was small to medium (*R^2^_marginal_* = 0.12, *R^2^_conditional_* = 0.23).

### Confusion index

The main effects of Vibration [*χ^2^*(1) = 26.45, *p* < 0.001, Figure 4B], and Emotion [*χ^2^*(1) = 319.32, *p* < 0.001, Supplementary Figure 4B] with absent vibrations associated with lower confusion compared to present vibrations [Absent: *m* = 9.37, *sd* = 21.6; Present: *m* = 13.9, *sd* = 27.4; *χ^2^*(1) = −3.84, *p* < 0.001], and joy associated with higher confusion compared to anger [Joy: *m* = 18.5, *sd* = 31.6; Anger: *m* = 5.28, *sd* = 13,2; *χ^2^*(1) = −13.01, *p* < 0.001]. The interactions between Emotion and Intensity [*χ^2^*(2) = 15.42, *p* < 0.001, Supplementary Figure 6B] and between Emotion and Vibration [*χ^2^*(1) = 17.14, *p* < 0.001, Supplementary Figure 5B] were significant as well. However, the main effect of Intensity [*χ^2^*(2) = 5.44, *p* = 0.07, Supplementary Figure 3B] only showed a tendency toward significance. Planned contrasts were performed to investigate the effect of Intensity and only a tendency toward significance was observed between low and medium intensities while the other comparisons were not significant [Low: *m* = 12.9, *sd* = 26.0; Medium: *m* = 11.0, *sd* = 24.4; High: *m* = 11.2, *sd* = 24.1; low vs. medium: *χ^2^*(1) = 2.01, *p* = 0.07]. The interaction between Intensity and Vibration [*χ^2^*(2) = 0.83, *p* = 0.66, Figure 5B] and the triple interaction [*χ^2^*(2) = 0.29, *p* = 0.86] were not significant. The effect size of this model was small to medium (*R^2^_marginal_* = 0.11, *R^2^_conditional_* = 0.23).

### Accuracy

The three main effects were significant [Vibration: *χ^2^*(1) = 19.69, *p* < 0.001, Figure 4C; Emotion: *χ^2^*(1) = 341.27, *p* < 0.001, Supplementary Figure 4C; Intensity: *χ^2^*(2) = 26.08, *p* < 0.001, Supplementary Figure 3C], with absent vibrations associated with higher accuracy (*m* = 0.83, *sd* = 0.31) compared to present vibrations [*m* = 0.78, *sd* = 0.36; *χ^2^*(1) = 0.04, *p* < 0.001], and anger associated with higher accuracy (*m* = 0.90, *sd* = 0.24) compared to joy [*m* = 0.71, *sd* = 0.40; *χ^2^*(1) = 0.19, *p* < 0.001]. Planned contrasts were performed to investigate the effect of Intensity and the comparison between low and medium intensities was significant [*χ^2^*(1) = −0.06, *p* < 0.001] with medium intensity associated with better accuracy than low intensity. The comparison between low and high intensities was significant as well [*χ^2^*(1) = −0.04, *p* < 0.01] while the other were not (Low: *m* = 0.77, *sd* = 0.36; Medium: *m* = 0.83, *sd* = 0.31; High: *m* = 0.81, *sd* = 0.33). The interaction between Emotion and Intensity was significant as well [*χ^2^*(2) = 18.53, *p* < 0.001, Supplementary Figure 6C]. However, the other double interactions [Emotion*Vibration: *χ^2^*(1) = 0.26, *p* = 0.61, Supplementary Figure 5C; Intensity*Vibration: *χ^2^*(2) = 1.91, *p* = 0.39, Figure 5C] were not significant, neither was the triple interaction [*χ^2^*(2) = 3.56, *p* = 0.17]. The effect size of this model was small to medium (*R^2^_marginal_* = 0.11, *R^2^_conditional_* = 0.21).

To investigate the impact of induced vibrations on perception levels and therefore test our hypotheses, we performed planned contrasts to test the effect of Vibration on the three indexes (discrimination, confusion and accuracy) and between the different levels of intensity but none of these comparisons were significant (Figures 4, 5). However, vibrations showed a significant effect on all three indexes when looking at each intensity levels separately. Taken together, results show an impact of Vibration—illustrating higher confusion and accuracy indices values, as well as lower discrimination index values with induced vibrations—but this effect never interacts with our other factors of interest, namely Emotion and Intensity.

## Discussion

The study of embodied cognition in the processing of emotional prosody is very poorly represented in the literature. This study aimed to investigate the influence of induced bodily vibrations in the perception of ambiguous emotional vocalizations. The main hypothesis was that the vibrations of the vocal cords—induced by vibrators—necessary for the production of vocalized sounds would constitute an interoceptive feedback that could influence the auditory perception of emotions. In accordance with the SIMS model (Niedenthal et al., 2010), it was also expected that these effects would be greater for stimuli that are more ambiguous. This study showed results contrary to our hypotheses, which can be explained by various limitations. Vibrations impacted the three indexes—discrimination, confusion and accuracy—with a tendency to confuse rather than facilitate vocal emotion processing.

Participants were presented with emotional voice stimuli expressing either anger or joy at three different acoustic intensities (low, medium, high), with vibrations expressing the same emotion induced in half of the trials. They had to evaluate each stimulus with four visual analogic scales (joy, anger, surprise, sadness). Three indices were computed from this evaluation: a discrimination index, a confusion index, and an accuracy index.

This study showed a significant effect of Vibration on all three indices (discrimination index, the confusion index and the accuracy index). However, the effect was in the opposite direction to the one expected. Indeed, the results showed that discrimination and accuracy indices were significantly higher when the vibrations were absent rather than present, and the opposite for the confusion index. We expected that vibrations, which were always congruent with emotional vocalizations, would have a facilitating effect on the recognition of these voice stimuli because participants would seek alternative sensory information, especially for low intensity or perceptually ‘ambiguous’ voices (Niedenthal et al., 2010). It seems that they brought instead global confusion to the participants. Although our results do not allow us to draw any conclusions, one potential explanation would be that individuals are not so good at interpreting their own emotions based on this interoceptive feedback of bodily resonances or more generally on their own vocalizations. Future research could integrate the Multidimensional Assessment of Interoceptive Awareness (MAIA; Mehling et al., 2012) to control for interindividual differences in interoceptive body awareness and its impact on the results. This would shed light on the interoceptive awareness in emotional voice perception and also production and pave the way to investigate more broadly its implication on the subjective feeling and therefore on emotion regulation. Indeed, some studies highlighted the impact of both interoceptive awareness and the intensity of emotional experiences (Wiens et al., 2000; Pollatos et al., 2007). It is also possible that the embodied simulation mechanism did not occur because the vibrations in this experiment did not constitute an interoceptive feedback since they were artificially induced. Indeed, perhaps the feedback that people get from the vibrations in the throat would only be informative when the vocalizations are produced by the own person. It is noteworthy that a study tested whether vocal simulation of melodies facilitate the induction of emotions in the context of music listening but did not find significant results (Cespedes-Guevara and Dibben, 2022). However, they manipulated explicit vocal and motor mimicry and not vibrations of the vocal tract, which could lead to other outcomes. Another possible explanation is that besides the fact that the vibrations may or may not have an effect, they represent primarily an unknown stimulation in the neck. This atypical sensation could capture the attention of the participants, leading to a reduction of their performance or a more general interference with the performed task. In fact, a study showed that participants can collect information from vibrotactile stimuli (Lylykangas et al., 2013) and another one presented mixed results on vibrotactile stimuli in emotion perception (Salminen, 2015). However, in both studies, participants had to focus on the vibration and not on another sensory modality as it is the case in our study with auditorily presented emotional voices. Nevertheless, it could also be hypothesized that there actually are motor simulations of vocal organs by the perceiver while processing the emotional vocalizations but that a conflict occurs between this body mimicry process of the perceiver and the induced vibrations, which masks the effect produced. Therefore, in future studies, it might be useful to place the vibrator in other body locations such as chest or wrist areas. Finally, we can point out that many researches on embodied cognition find small or no effect at all, or even fail to be replicated (Strack et al., 1988; Cespedes-Guevara and Dibben, 2022). This could be explained by the “weak” embodiment theories, stating that sensorimotor representations are only a part of high-level cognition (Meteyard et al., 2012) and that their effect can therefore be masked by other processes. This mechanism proved to be delicate to study and we hopefully will be able to explore it with different paradigms in further studies. On a more peripheral level, we can see in Supplementary Table 1 that anger was better discriminated from other emotions, namely more accurately recognized and less confused with other emotions in contrast to joyful voices. It follows the study of Petrushin (2000), who also found that amongst several emotions, anger was discriminated the best, or other studies showing a high average accuracy of anger recognition (Sander et al., 2005; Bänziger et al., 2009).

Our secondary hypothesis was that the vibrations would have a greater effect specifically for the most ‘ambiguous’ stimuli—namely, voices with the lowest acoustic intensity—as supported by the SIMS model (Niedenthal et al., 2010) for facial expressions. Indeed, in our study, ‘ambiguous’ stimuli were represented by low intensity as a more perceptual ambiguity, as opposed to medium and high intensity voices, considered as less to non-ambiguous stimuli. Interaction between Vibration and Intensity did not turn out to be significant, and the planned contrasts performed to further investigate this interaction were not significant either. These results are in contradiction with our interaction hypothesis mentioned above, and therefore with the SIMS model.

## Limitations

Our study has some limitations that would partly explain the results we obtained. First, our sample is mostly composed of female participants whereas some studies showed gender differences in recognition of emotional prosody (Imaizumi et al., 2004; Lambrecht et al., 2014) and when using facial mimicry (Dimberg and Lundquist, 1990; Niedenthal et al., 2012; Korb et al., 2015). In the same way, our sample is mostly composed of psychology students. Both of these elements limit the generalizability of our results to a general population. On another part, the resolution of the vibrator is low compared to the frequency domain of the human voice, which leads to some important spectro-temporal elements of the emotion not being conveyed properly from the voice stimuli. Several studies demonstrated the role of acoustic properties in the auditory recognition of emotions and especially the importance of spectral cues (Banse and Scherer, 1996; Grandjean et al., 2006; Sauter et al., 2010). However, the vibrator has a sensitivity of 1 kHz, which is well below the frequency resolution of human voice—meaning that there was a loss of transmitted information which could impair the mechanism of interoceptive feedback mediated by the vibrator and may be the source of this result. Nevertheless, as mentioned before, the frequency of the bodily resonances measured by Sundberg (1992) corresponds mainly to the fundamental frequency of the tone produced, which suggests that this might not be so much of a problem. A study with the same equipment specifically controlling the perception of the vibrations would be relevant for further investigations. Or, in the same direction, it could be interesting to test the influence of several mere F0 frequencies in this kind of task. Moreover, some participants reported hearing the sound of the vibrator. Indeed, the device emitted the vibrations but also a weak residual sound. This could contribute to the global confusion that was observed with the presence of the vibrations and it could have distracted the participants from the main task as well. Second, we expected the recognition of voice stimuli in response to the intensity of the voice stimuli to be linear but it seems that “Low Intensity” and “High Intensity” modalities are not optimal for the recognition and were both less well recognized than the “Medium Intensity” voices (Supplementary Figure 3). Our goal was to compare the “Low Intensity” with the two others to investigate the perceptual ambiguity but it seems that testing ambiguity with these stimuli is problematic. Perhaps this kind of ambiguity is not suitable for initiating the process of seeking additional sources of information such as interoceptive feedback. A future study should test another kind of ambiguity of the voice stimuli or use finer intensities. For example, an emotional ambiguity could be tested by morphing voice stimuli expressing two distinct emotions. Such voice stimuli could perhaps further involve the use of interoceptive feedback or at least remove some interference from induced vibrations.

## Conclusion

In conclusion, it would seem that manipulating the intensity of emotional voice stimuli is not an adequate way to assess the impact of the bodily resonances as an interoceptive feedback that could influence the auditory perception of emotions. Further investigations are needed to explore this mechanism more deeply, for instance by using other vocal emotions, paradigms and stimuli, vibrator locations, or trying to compensate for low vibrator spatiotemporal resolution. Our data pave the way to other studies in the field of embodied cognition in the context of vocal emotion perception, as we believe this is a promising area of research.

## Data availability statement

The raw data supporting the conclusions of this article will be made available by the authors, without undue reservation.

## Ethics statement

The studies involving human participants were reviewed and approved by Ethics Committee of the University of Geneva. The patients/participants provided their written informed consent to participate in this study.

## Author contributions

All authors listed have made a substantial, direct, and intellectual contribution to the work and approved it for publication.

## Funding

This project was part of a fund granted by the Swiss National Science Foundation (grant number UN10774 to DG and LC). Open access funding was provided by the University of Geneva.

## Conflict of interest

The authors declare that the research was conducted in the absence of any commercial or financial relationships that could be construed as a potential conflict of interest.

## Publisher’s note

All claims expressed in this article are solely those of the authors and do not necessarily represent those of their affiliated organizations, or those of the publisher, the editors and the reviewers. Any product that may be evaluated in this article, or claim that may be made by its manufacturer, is not guaranteed or endorsed by the publisher.
