# Supplementary material for: Influence of bodily resonances on emotional prosody perception
Source: Front Psychol. 2022 Dec 8;13:1061930. doi: 10.3389/fpsyg.2022.1061930 (PMC9773097; doi:10.3389/fpsyg.2022.1061930)
Supplement: Supplementary file 1 [file Data_Sheet_1.docx]

**SUPPLEMENTARY DATA**

# Supplementary Figures


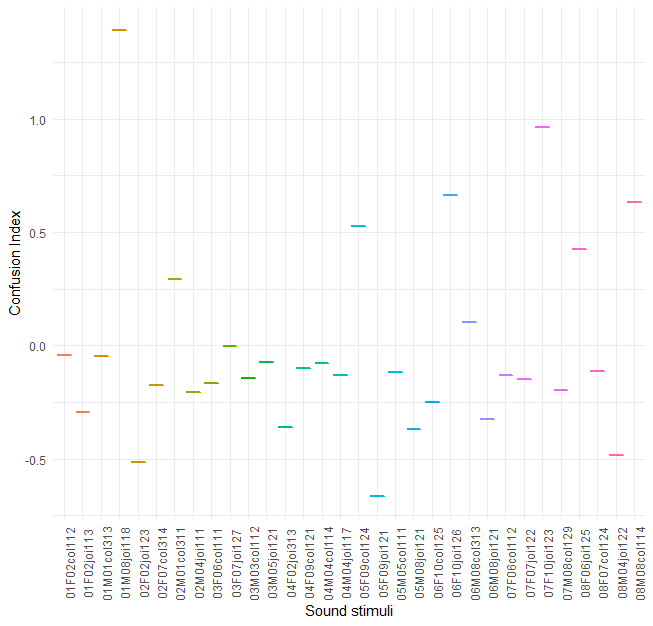

***Supplementary Figure 1.* Z-scores of the confusion index for each sound.** Graph showing the z-scores of the confusion index for each sound. The fourth one (01M08joi118) was excluded after a central limit theorem which showed that it was an extreme value (*Z* = 3.96, *P* < .001).


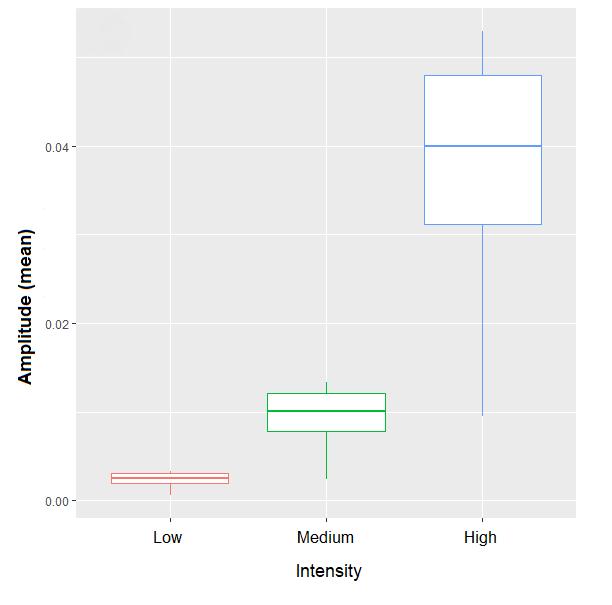

***Supplementary Figure 2.* Boxplot showing the variation in amplitude as a function of stimulus intensity.** On the X axis, each point represents an Intensity condition (i.e., low, medium and high).


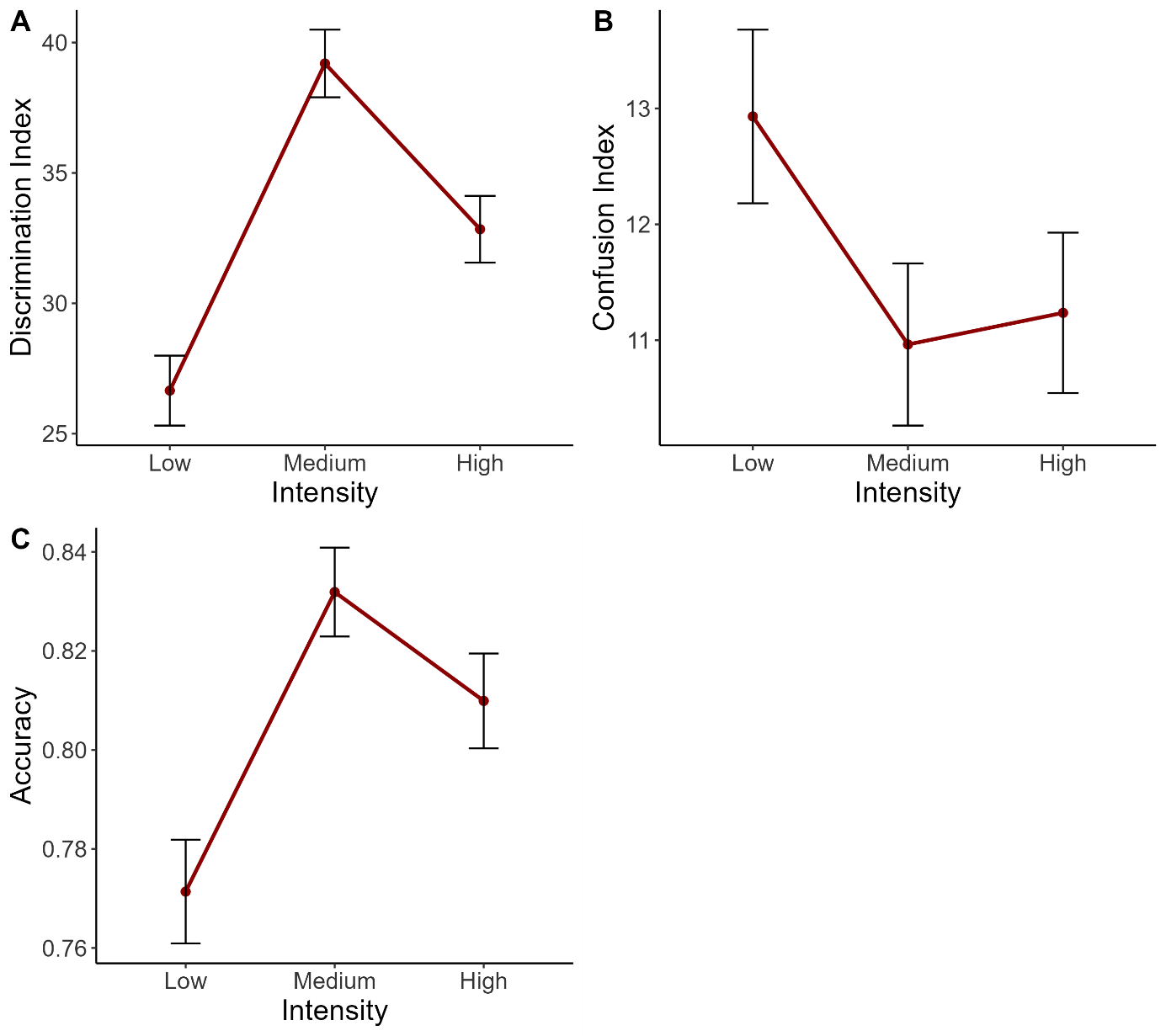

***Supplementary Figure 3.* Main effect of Intensity on A) the discrimination index, B) the confusion index and C) the accuracy.** On the X axis, each point represents an Intensity condition (i.e., low, medium and high). Error bars indicate the standard error of the mean.


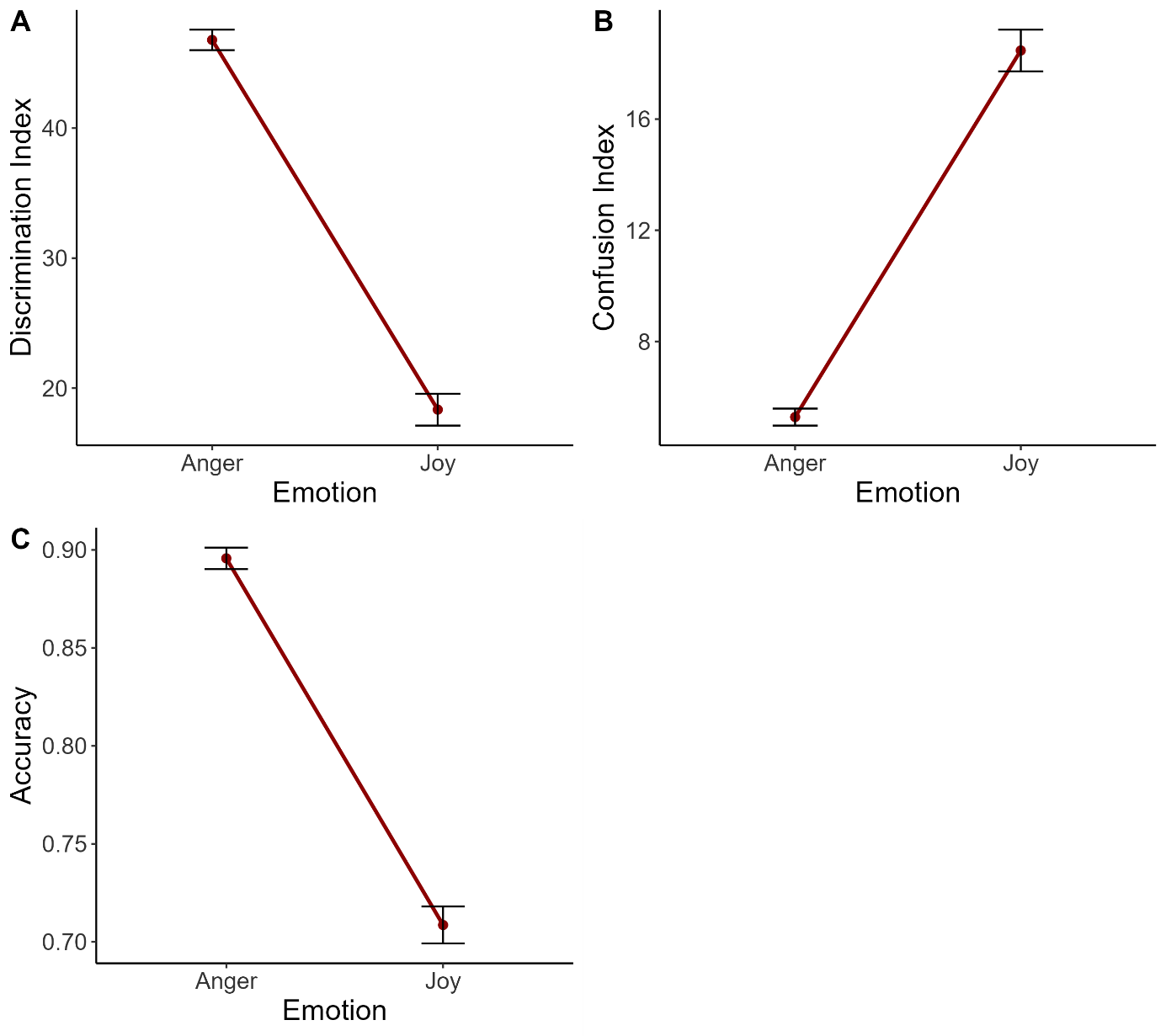
***Supplementary Figure 4.* Main effect of Emotion on A) the discrimination index, B) the confusion index and C) the accuracy.** On the X axis, each point represents an Emotional vocalization condition (i.e., anger vs joy). Error bars indicate the standard error of the mean.


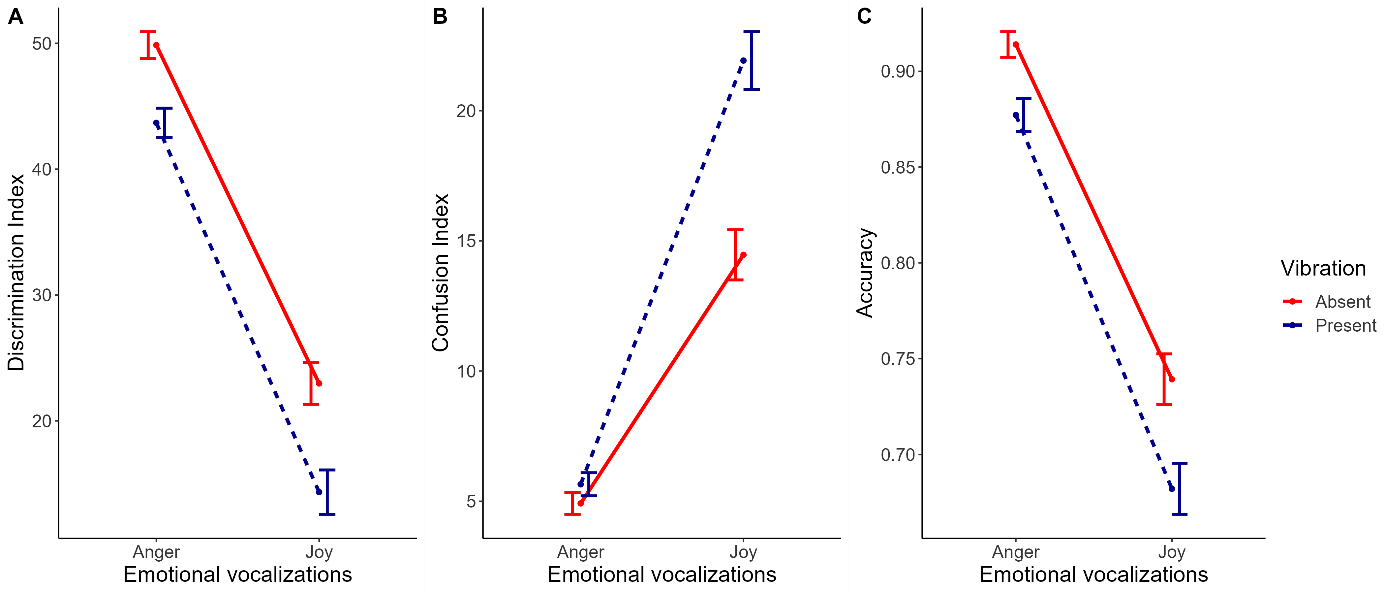
***Supplementary Figure 5.* Interactions between Vibration and Emotion on A) the discrimination index, B) the confusion index and C) the accuracy.** On the X axis, each point represents an Emotional vocalization condition (i.e., anger vs joy). Each line represents a Vibration condition (i.e., absent vs present). Error bars indicate the standard error of the mean.


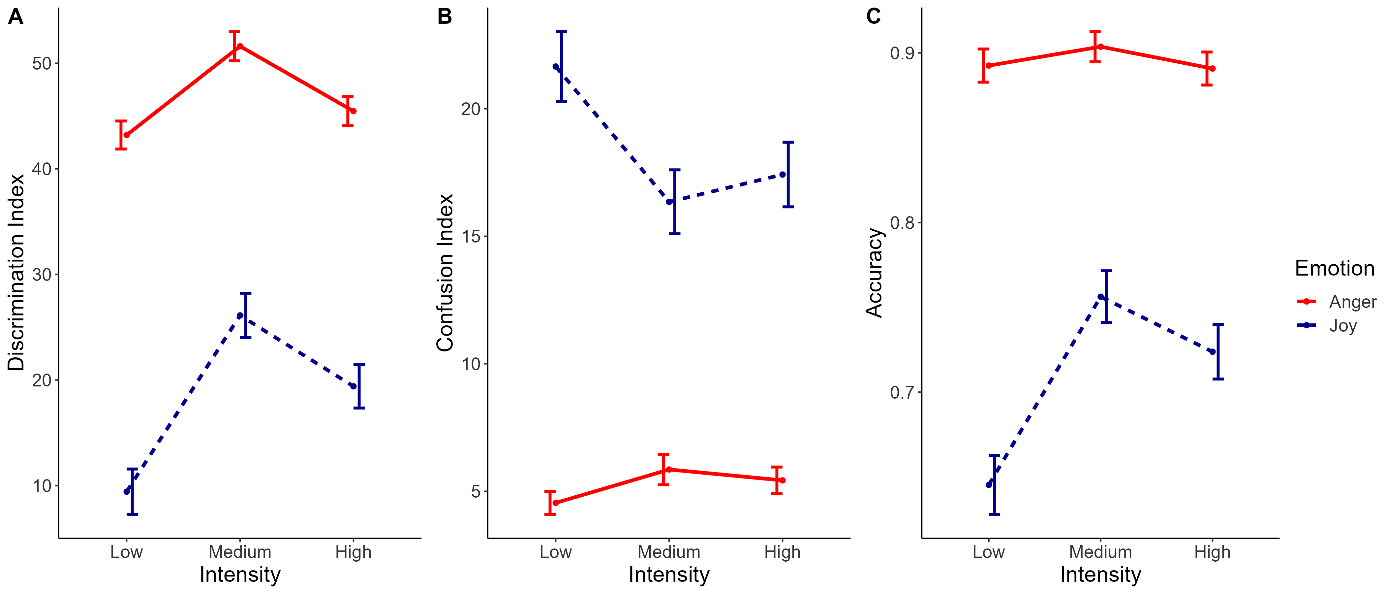
***Supplementary Figure 6.* Interactions between Intensity and Emotion on the mean of A) the discrimination index, B) the confusion index and C) the accuracy.** Intensity conditions are represented on the X axis (i.e., low, medium and high). Each line represents an Emotional vocalization condition (i.e., joy vs anger). Error bars indicate the standard error of the mean.

# Supplementary tables

**Supplementary Table 1***Confusion matrix: Number (percentage) of each emotion was marked as dominant emotion respectively for anger and joy voice stimuli*

|  | **Anger voice stimuli** | **Joy voice stimuli** |
| --- | --- | --- |
| Anger | 1725 (92.39) | 48 (2.71) |
| Joy | 21 (1.12) | 1303 (73.49) |
| Fear | 88 (4.71) | 197 (11.11) |
| Sadness | 33 (1.77) | 225 (12.69) |

**Supplementary Table 2**
*Results of the analysis of acoustic parameters as a function of emotion and intensity*

|  |  | **Fisher** | | |  | **Effect sizes** | |
| --- | --- | --- | --- | --- | --- | --- | --- |
| **Variables** | **Factors** | ***df1*** | ***df2*** | ***F-value*** | ***P*** | ***R^2^_marginal_*** | ***R^2^_conditional_*** |
| Amplitude | Emotion | 1 | 95 | .34 | .56 | .86 | .90 |
|  | Intensity | 2 | 94 | 393 | <.001 |  |  |
| Loudness | Emotion | 1 | 95 | 1.93 | .17 | .77 | .80 |
|  | Intensity | 2 | 94 | 180.23 | <.001 |  |  |
| Pitch | Emotion | 1 | 95 | 7.36 | <.01 | .05 | .35 |
|  | Intensity | 2 | 94 | .00 | ~.99 |  |  |
| Roughness | Emotion | 1 | 95 | 10.58 | <.01 | .09 | .17 |
|  | Intensity | 2 | 94 | .00 | ~.99 |  |  |
| Spectral Centroid | Emotion | 1 | 95 | 8.56 | <.01 | .10 | .10 |
|  | Intensity | 2 | 94 | 1.12 | .33 |  |  |
| Spectral Slope | Emotion | 1 | 95 | 56.81 | <.001 | .36 | .40 |
|  | Intensity | 2 | 94 | .00 | ~.99 |  |  |
| F1 frequency | Emotion | 1 | 95 | 5.33 | <.05 | .06 | .11 |
|  | Intensity | 2 | 94 | .27 | .76 |  |  |
| F2 frequency | Emotion | 1 | 95 | 15.79 | <.001 | .14 | .15 |
|  | Intensity | 2 | 94 | .15 | .86 |  |  |

*Linear Mixed Models (LMM) were performed to analyze the acoustic parameters above, with two factors: Emotion (joy, anger) and Intensity (low, medium, high), and a random effect: actor’s gender (male, female).*

**Supplementary Table 3**
*Results of the analysis of emotion-specific confusion indices as a function of emotion, intensity and vibration*

|  |  | **Fisher** | | |  | **Effect sizes** | |
| --- | --- | --- | --- | --- | --- | --- | --- |
| **Variables** | **Factors** | ***df1*** | ***df2*** | ***F-value*** | ***P*** | ***R^2^_marginal_*** | ***R^2^_conditional_*** |
| Anger CI | Intensity | 2 | 19 | 31.57 | <.001 | .04 | .05 |
|  | Vibration | 1 | 20 | 2.06 | .15 |  |  |
| Joy CI | Intensity | 2 | 19 | 35.65 | <.001 | .05 | .06 |
|  | Vibration | 1 | 20 | 34.85 | <.001 |  |  |
| Fear CI | Emotion | 1 | 20 | 2.75 | .10 | .04 | .04 |
|  | Intensity | 2 | 19 | 54.89 | <.001 |  |  |
|  | Vibration | 1 | 20 | 27.13 | <.001 |  |  |
| Sadness CI | Emotion | 1 | 20 | 12.61 | <.001 | .05 | .06 |
|  | Intensity | 2 | 19 | 66.90 | <.001 |  |  |
|  | Vibration | 1 | 20 | 27.12 | <.001 |  |  |

*Note: CI = confusion index. For each trial, emotion-specific confusion indices were computed. As four responses were permitted (joy, anger, fear, sadness), 4 indices were calculated following this example with the sadness confusion index: ScoreResponseSadness – ScoreExpressedEmotion. Therefore, for each index, we obtain a positive score if the emotion is rated higher than the expressed emotion, indicating a higher confusion in relation to this emotion, and negative if the expressed emotion is rated higher, indicating a lower confusion. Since for joy and anger, both response and expressed emotion are the same for half of the trials, the two indices were coded non available (“NA”) in these cases as it does not give information about confusion. For the same reason, Emotion was not included as a factor in their analyses. LMMs were performed to analyze each emotion-specific confusion indices as a function of Intensity (low, medium, high), Vibration (present, absent) and Emotion (joy, anger) only for the fear and sadness specific confusion indices. The following random effects were integrated: participant’s identifier, participant’s gender, actor’s gender and block number. Only main effects were included in this table.*
